# Supplementary material for: Comparison of a New 68Ga-Radiolabelled PET Imaging Agent sCD146 and RGD Peptide for In Vivo Evaluation of Angiogenesis in Mouse Model of Myocardial Infarction
Source: Cells. 2021 Sep 3;10(9):2305. doi: 10.3390/cells10092305 (PMC8466330; doi:10.3390/cells10092305)
Supplement: Supplementary file 1 [file cells-10-02305-s001.zip › cells-1338355-supplementary.pdf]

Supplementary data:

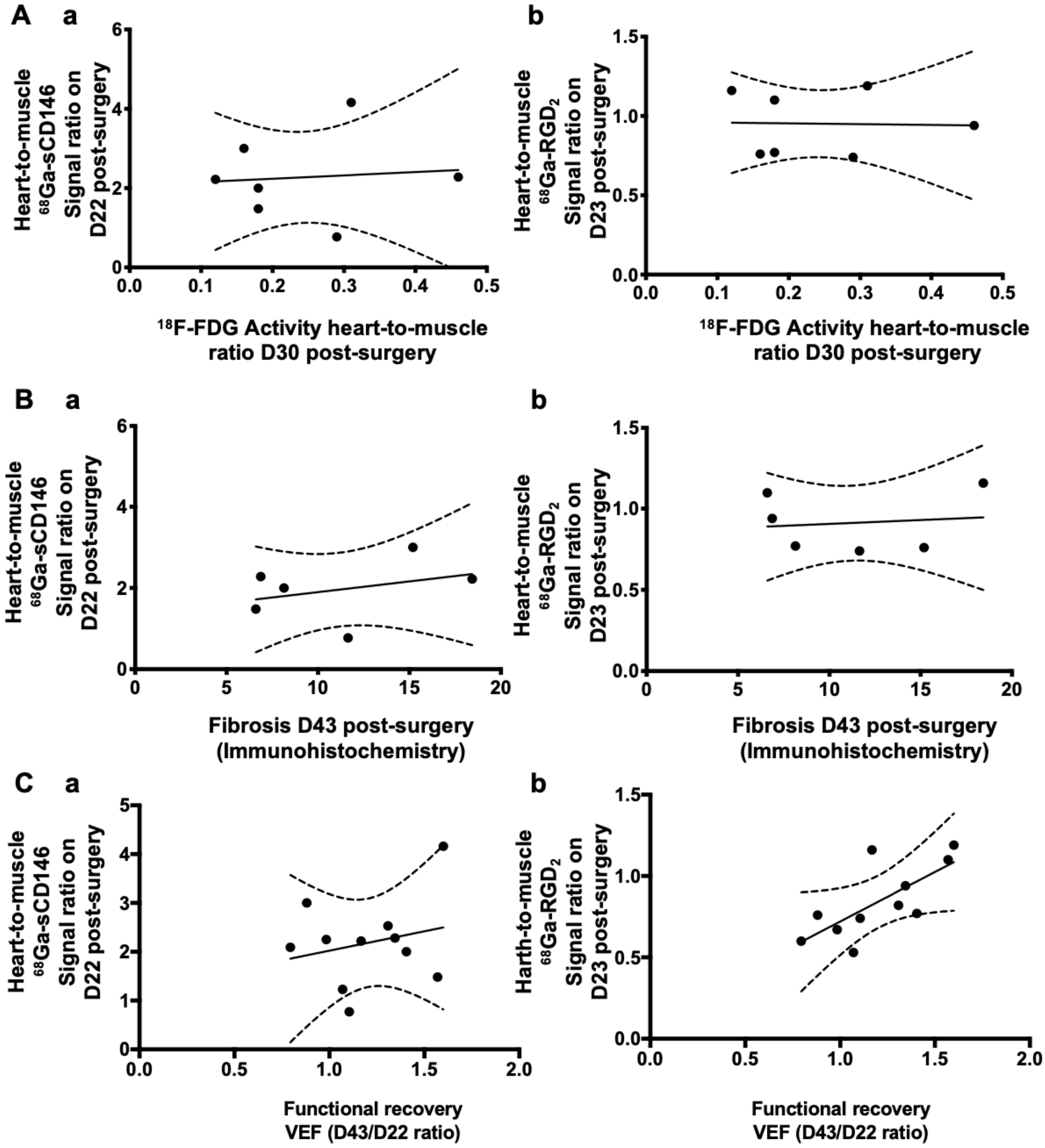

Figure S1. Correlation analysis. (**Aa**) No significant positive correlation between residual perfusion recovery perfusion recovery evaluated by ^{18}F-FDG microPET signal expressed as heart-to-muscle ratio on day 30, and ^{68}Ga-sCD146 microPET signal intensity expressed as heart-to-muscle ratio on day 22 post-myocardial infarction (Pearson R^2=0.09; P = 0.42; n = 7). (**Ab**) No significant correlation was observed between individual residual perfusion recovery and ^{68}Ga-RGD$_2$ microPET signal intensity on day 23 post-ischemia (Pearson R^2 = -0.02; P = 0.47; n = 7). (**Ba**): No Significant correlation was detected between individual fibrosis quantification evaluated by histological Sirius red staining and expressed as percentage of fibrosis tissue on left ventricle 43 days post-myocardial infarction, and ^{68}Ga-sCD146 microPET signal intensity expressed as heart-to-muscle ratio on day 22 post-ischemia (Pearson R^2 = 0.33; P = 0.25; n = 6). (**Bb**) No significant correlation was observed between individual fibrosis damages on day 43 post-ischemia, and ^{68}Ga-RGD$_2$ PET signal on day 23 post-ischemia (Pearson R^2 = 0.12; P = 0.40; n = 6). (**Ca**) No significant correlation was found between individual functional recovery explored with ultrasound imaging and expressed as the ratio between VEF 43 and 22 days post-myocardial infarction and ^{68}Ga-sCD146 microPET signal intensity expressed as heart-to-muscle ratio on day 22

post-myocardial infarction (Pearson $R^2 = 0.23$; $P = 0.48$; $n = 11$). (**Cb**)A significant correlation is identified between individual functional recovery and ^{68}Ga-RGD$_2$ microPET signal intensity on day 23 post-ischemia (Pearson $R^2 = 0.72$; $P = 0.01$; $n = 11$).

		^{18}F-FDG PET signal D7	^{68}Ga-SCD146 PET signal D15	^{68}Ga-RGD PET signal D14	^{68}Ga-SCD146 PET signal D22	^{68}Ga-RGD PET signal D23	histological Sirius red staining intensity	VEF (D43/D22 ratio) ultrasound imaging
IM mice	Mean	0,17	3,23	3,03	2,27	0,95	11,15	1,30
	Sd	0,13	0,83	1,57	1,08	0,20	4,85	0,26
Sham mice	Mean	1,01	2,03	1,64	2,03	0,66		1,04
	Sd	0,22	0,26	0,81	0,56	0,12		0,21

Table S1. Semi-quantitative analysis of PET imaging analysis, histological Sirius red intensity and ultrasound imaging expressed on Mean and Standard deviation for IM mice and Sham mice.
